# Prevalence of medication errors and its related factors in Iranian nurses: an updated systematic review and meta-analysis

**DOI:** 10.1186/s12912-024-01836-w

**Published:** 2024-03-14

**Authors:** Hadis Fathizadeh, Samaneh-Sadat Mousavi, Zahra Gharibi, Hamidreza Rezaeipour, Abdol-Rahim Biojmajd

**Affiliations:** 1 Department of Laboratory Sciences, Sirjan School of Medical Sciences, Sirjan, Iran; 2 Student Research Committee, Sirjan School of Medical Sciences, Sirjan, Iran; 3 Infectious and Tropical Diseases Research Center, Hormozgan Health Institute, Hormozgan University of Medical Sciences, Bandar Abbas, Iran; 4 Department of Nursing, Sirjan School of Medical Sciences, Sirjan, Iran

**Keywords:** Prevalence, Nurses, Medication errors, Reporting rate, Iran, Systematic review, Meta-analysis

## Abstract

**Background:**

Nurses may make medication errors during the implementation of therapeutic interventions, which initially threaten the patient’s health and safety and prolong their hospital stay. These errors have always been a challenge for healthcare systems. Given that factors such as the timing, type, and causes of medication errors can serve as suitable predictors for their occurrence, we have decided to conduct a review study aiming to investigate the prevalence of medication errors and the associated factors among Iranian nurses.

**Methods:**

In this systematic review and meta-analysis, studies were searched on PubMed, Web of Science, Scopus, Google Scholar, IranMedex, Magiran, and SID databases using a combination of keywords and Boolean functions. The study that reported the prevalence of medication errors among nurses in Iran without time limitation up to May 2023 was included in this study.

**Results:**

A total of 36 studies were included in the analysis. The analysis indicates that 54% (95% CI: 43, 65; I2 = 99.3%) of Iranian nurses experienced medication errors. The most common types of medication errors by nurses were wrong timing 27.3% (95% CI: 19, 36; I2 = 95.8%), and wrong dosage 26.4% (95% CI: 20, 33; I2 = 91%). Additionally, the main causes of medication errors among nurses were workload 43%, fatigue 42.7%, and nursing shortage 38.8%. In this study, just 39% (95% CI: 27, 50; I2 = 97.1%) of nurses with medication errors did report their errors. Moreover, the prevalence of medication errors was more in the night shift at 41.1%. The results of the meta-regression showed that publication year and the female-to-male ratio are good predictors of medical errors, but they are not statistically significant(*p* > 0.05).

**Conclusions:**

To reduce medication errors, nurses need to work in a calm environment that allows for proper nursing interventions and prevents overcrowding in departments. Additionally, considering the low reporting of medication errors to managers, support should be provided to nurses who report medication errors, in order to promote a culture of reporting these errors among Iranian nurses and ensure patient safety is not compromised.

**Supplementary Information:**

The online version contains supplementary material available at 10.1186/s12912-024-01836-w.

## Introduction

Patient safety is considered a crucial issue in today’s world, and it is used as a valuable indicator for controlling the quality of healthcare services provided by healthcare professionals [1–4]. Despite technological advancements in the healthcare system, medical errors continue to pose a threat to patient safety. For instance, the implementation of electronic systems for medication ordering and recording, as well as the use of automated devices for medication administration and dosage determination, represent some of the technological advancements that can be utilized in this regard [5, 6]. Medical errors are one of the most common hazards in patient care and hospital safety. Approximately 10% of hospitalized patients are confronted with medical errors, and 7% of these errors result in mortality [7]. Medication errors are one of the most common types of medical errors and are classified into five categories by the American Medical Association [8]. These errors include medication errors, errors in conducting laboratory tests, errors in filing, errors in providing services, and errors in response to abnormal results of experimental tests [9]. The annual statistics of mortality due to medical errors indicate that 98,000 individuals die as a result of these errors. Among these cases, 7,000 are solely attributed to medication errors [10].

Medication errors can occur at various stages of the medication prescribing process. However, common medication errors include prescribing the wrong medication, not adhering to the correct timing of medication administration, not following the appropriate route of medication administration, prescribing medication in a dosage exceeding the prescribed order, administering medication to the wrong patient, and miscalculating medication dosages [11–13]. Adverse effects resulting from medication errors impose costs equivalent to 11 billion dollars annually on governments [14]. To prevent medication errors, it is important to carefully follow eight principles: ensuring the correct patient, administering the correct drug at the correct time and route, providing the correct dose, accurately documenting, prescribing appropriately, and monitoring the patient’s response to the medication [15].

Administering medication to patients is the primary and important responsibility of nurses, and it is referred to as the main responsibility of nurses. Approximately 40% of nurses’ time in each shift is spent on performing this task, resulting occurrence of medication errors among nurses [16].The reasons for the occurrence of medication errors by nurses can be attributed to rapid advancements in medical technology, existing deficiencies in nursing education, and high public expectations of nurses, which lead to significant stress on nurses and create a conducive environment for errors to occur [17, 18].

The rate of medication errors reported by nurses is significantly lower than reality [19, 20]. In a study conducted in Iran, only 19% of nurses reported their medication errors, which leads to compromised patient safety and increased hospitalization and treatment duration for patients [21]. However, the prevalence of medication errors has significantly increased due to the wide range of medications and the growing number of patients taking multiple drugs, despite the availability of guidelines [22].

Numerous and diverse studies have been conducted on medication errors. The first step in solving a problem is understanding its prevalence and extent. In a recent study conducted by Matin, [23] factors such as the type of medication error, shift occurrence of errors, causes of medication errors, and the impact of the questionnaire used, population size, and number of hospitals on the occurrence of medication errors by nurses were not investigated. Considering that these factors can serve as appropriate predictors for medication errors by nurses in hospital settings for healthcare and medical system managers worldwide, and also considering the numerous related articles published since the publication of a similar article, conducting an up-to-date systematic review and meta-analysis can be helpful.

## Methods

This study was a systematic review and meta-analysis approved by the Research and Technology Committee of the Student Research Center at Sirjan School of Medical Sciences (No: 402,000,004) and has an ethics code from the Ethics Committee for Biomedical Research at Sirjan School of Medical Sciences (NO: IR.SIRUMS.REC.1402.004). This study aims to investigate the prevalence of medication errors among Iranian nurses based on published articles in domestic and international journals through a systematic review and meta-analysis approach until the end of May 2023. The present study is designed based on the PRISMA checklist [24].

Two researchers(AB.B, H.F) conducted searches on scientific databases. In this study, national and international databases were searched. These databases included: PubMed, Web of Science, Scopus, Google Scholar, IranMedex, Magiran, and SID. All relevant articles published in Persian or English regarding medication errors among Iranian nurses were searched. Additionally, the references of the final studies were searched to find other related articles. The following keywords were used in combination:(‘medication error(s)’ OR “Drug use error” OR “wrong drug” OR “wrong dose” OR ”prescribing error(s)” OR ‘drug error(s)’ OR ‘medication mistake(s)’ OR “dispensing error“ OR “incorrect drug” OR “incorrect dose” OR “administration error” OR ‘drug mistake (s)’ OR “transcription error” OR “inappropriate prescribing” OR “inappropriate medication”) AND(‘nurse(s)’ OR ‘nursing staff’) AND (‘Iran’ OR ‘Iranian’).

### Study selection

The following four inclusion criteria were used: [1] articles published in Persian or English language; [2] articles published from the beginning until May 2023; [3] research conducted in a hospital setting; [4] cross-sectional studies that reported the prevalence of medication errors among nurses over a lifetime or a period of time. The following two exclusion criteria were used Studies have investigated medication errors among students, physicians, pharmacists, and other healthcare professionals. All studies were independently evaluated by two authors(AB.B and H.R) and any discrepancies were resolved through discussion and consultation with a third author(H.F), if necessary.

### Data extraction and quality assessment

In this study, a checklist designed by the authors was used to extract information. The extracted information from each study included: first author, year of publication, year of data collection, gender ratio, language of the study, average age, average work experience, sample size, number of hospitals, number of medication errors, study province, and other relevant information related to medication errors. Two independent researchers extracted data from each article (AB.B and H.R).

We used the critical appraisal checklist developed by the Joanna Briggs Institute (JBI) to evaluate the quality of the studies included in our research [25]. This checklist is specifically designed for cross-sectional studies and comprises 8 domains. A score of more than 6 indicates high quality, scores ranging from 3 to 6 indicate moderate quality, and scores below 3 represent low-quality studies. It’s important to note that we did not exclude any studies based on their quality score. Two investigators independently assessed the methodological quality of primary studies. In case of uncertainty or disagreement between reviewers, an independent investigator was consulted to reach a consensus (AB.B, F.S and H.F).

### Statistical analysis

In the current study, the prevalence of medication errors was obtained as the ratio of nurses who reported their own medication errors to the total number of nurses who experienced medication errors during a specific period or throughout their career. In this study we computed the standard error (SE) of the prevalence of medication errors among nurses and the prevalence of reporting medication error to nurse managers. The I^2^ (statistical measure that represents the degree of heterogeneity) index was used to assess the heterogeneity of data, which was categorized as low heterogeneity (I^2^ index < 25%), average heterogeneity (I^2^ index = 25–75%), and high heterogeneity (I^2^ index > 75%). Considering the heterogeneity index (I^2^) which was more than 75%, as well as the significance of Cochran’s Q (*p* < 0.0001). In this study, data were analyzed using a random effects model due to the high heterogeneity (I^2^ > 75%) [26, 27]. Subgroup analysis was conducted to evaluate the prevalence of MEs based on factors such as shift work, Type of medication error, cause of MEs Number of error reports, and others. Additionally, meta-regressions were performed based on age, work experience, year of publication, and gender ratio of female to male. Also, Egger’s test were used to assess publication bias and the influence of small studies. STATA software (version 17) was utilized for data analysis.

## Result

**Fig. 1:**
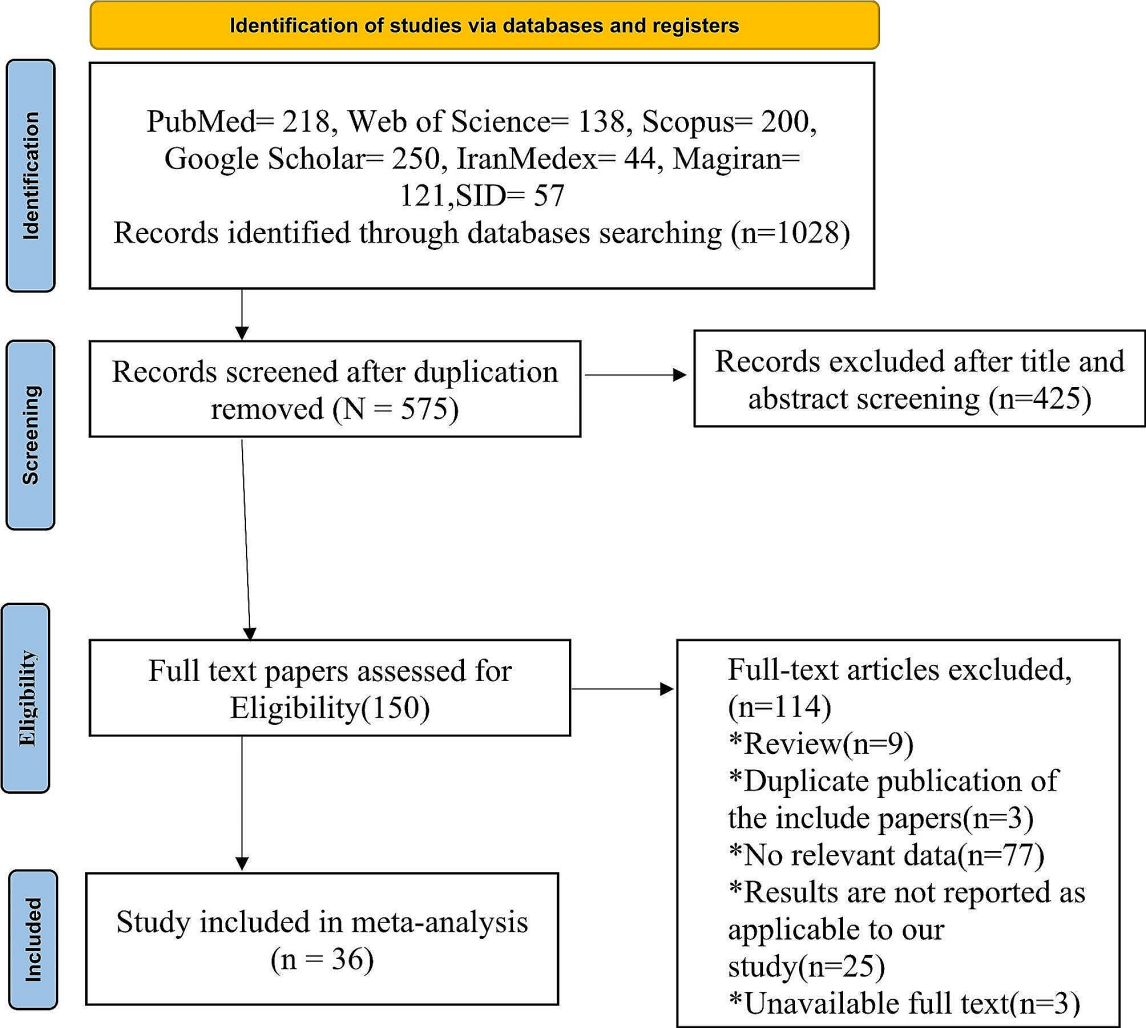
The PRISMA flow diagram showing the study selection process

Initially, we identified a total of 1028 reports, which we then examined for any duplicates and evaluated based on their respective titles and abstracts. Following this review, we excluded any duplicates (453 article) and reduced the number of reports to 575. Next, we further assessed these 575 reports to determine their eligibility, resulting in 150 studies that met our inclusion criteria and underwent a secondary evaluation. Finally, after all, evaluations were complete, we included 36 studies in our final analysis Fig. 1. Reasons for exclusion some study were unrelated topic and unrelated study population.

A total of 36 articles were analyzed in the meta-analysis, encompassing 6,238 participants with an average of 137.28 individuals per study. The largest and smallest sample sizes were reported in studies by Fathi [28] (500 participants) and by Fathi [29] (40 participants), respectively. Table 1 presents the general characteristics of the chosen studies Table 1.

**Table 1:** Characteristic of the included articles

First author	Year of data collection	Age	Experience	N (Total)	Prevalence (MEs)	Prevalence of report MEs	N(hospital)	Female/male	Quality	United
Salmani et al [52]	2015	31.5	6.8	71	47.9	-	5	-	Medium	Yazd
Cheraghi et al [59]	2011	33	4	64	73.4	42.5	1	8.1	Medium	Tehran
Mirzaei et al [48]	2012	31.1	-	96	79.2	28.9	1	2.7	Medium	Kermanshah
Pournamdar et al [60]	2016	28.9	-	119	72.3	-	-	5.6	Medium	Zahedan
Fathi et al [28]	2016	31.9	5.5	500	17	55	7	1.9	High	Kermanshah
Salmani et al [36]	2015	32.7	-	63	71.4	-	-	-	Low	Yazd
Cheragi et al [61]	2009	-	-	237	64.5	42.5	1	2	Medium	Tehran
Piroozi et al [37]	2016	-	5.6	366	10	-	6	2.8	High	Kurdistan
Miladinia et al [62]	2014	31	6.4	53	58.5	-	5	3.4	Low	Ahwaz
Yeke zaree et al [35]	2015	31.7	-	379	29	-	16	-	High	Tehran
Saremi et al [34]	2012	38.3	13.3	150	34.8	-	1	-	Medium	Tehran
Mohammad-Nejad et al [63]	2011	27.7	7.3	94	44.6	27.3	1	6.8	Medium	Tehran
Shams et al [64]	2011	34.5	-	350	28.9	14.4	-	2.8	High	West-Azarbaijan
Musarezaie et al [65]	2012	-	-	280	20	13.6	-	5.6	High	Isfahan
Ebrahimpour et al [66]	2013	30.7	8.2	150	40.8	82	4	6.5	Medium	Qazvin
Taheri et al [33]	2011	31.9	-	119	88.2	-	5	-	Medium	Tehran
Ramazani et al [32]	2014	31.6	-	180	80	-	10	-	Medium	Yazd
Gholipour et al [67]	2014	30.3	5	120	85.8	-	1	8.2	Medium	West-Azarbaijan
Farzi et al [1]	2014	36	10.7	235	80	72	-	2.8	High	Isfahan
Mosakazemi et al** [68]	2016	27	-	106	72.6	10.6	2	2.1	Medium	Shiraz
Farajzadeh et al [40]	2018	31	7.7	210	49.6	-	1	1.8	Medium	Kurdistan
Geravandi et al** [69]	2014	-	-	88	60	-	1	7.8	Medium	Ahwaz
Sabzi et al [31]	2017	-	-	91	59.6	-	1	-	Medium	Golestan
Mirzaei-Alavijeh et al [70]	2013	29.7	7	58	22.4	-	6	0.7	Low	Kermanshah
Bagheri et al [49]	2017	34	10.7	105	33.3	-	-	2.2	Medium	Yazd
Dehvan et al [71]	2015	-	-	56	69.5	-	-	13	Medium	Semnan
Dashti et al [21]	2017	33	8.7	191	86.4	19.4	-	22.9	Medium	Ardabil
Sharbaafchi zadeh et al [72]	2018	37.1	12.5	220	66.8	75.6	1	1.4	Medium	Isfahan
Sarhadi et al [73]	2014	31.2	8	400	28	16	2	4.1	High	Zahedan
Ahangarzadeh-Rezae et al. [38]	2010	29.5	-	100	100	-	-	1.4	Medium	Kurdistan
Ghorbanpour Diz et al [30]	2015			148	76	28	2	-	Medium	Tehran
Derikvand et al [74]	2017	-	-	294	69	52.2	-	1.8	High	Khoramabad
Fathi et al [29]	2014	-	-	40	30.3	-	1	4.8	Low	Tehran
Penjvini et al [75]	2001	-	-	104	16.7	-	-	3	Medium	Kurdistan
Ghannadi et al [76]	2011	35.2	10.3	65	48.2	-	1	4	Medium	Khoramabad
Ghobadi et al [77]	2020	-	-	336	33.3	32.6	3	3.5	Medium	Tehran

The majority of respondents in our study (73.3% of total respondents) were female, although seven author teams did not report gender of respondents [30–36]. The average age of nurses, based on 25 studies that reported the average age, was determined to be 32.6 years. In addition, the average work experience of nurses was obtained as 7.97 years based on 17 studies reporting the work experience of nurses.

**Fig. 2:**
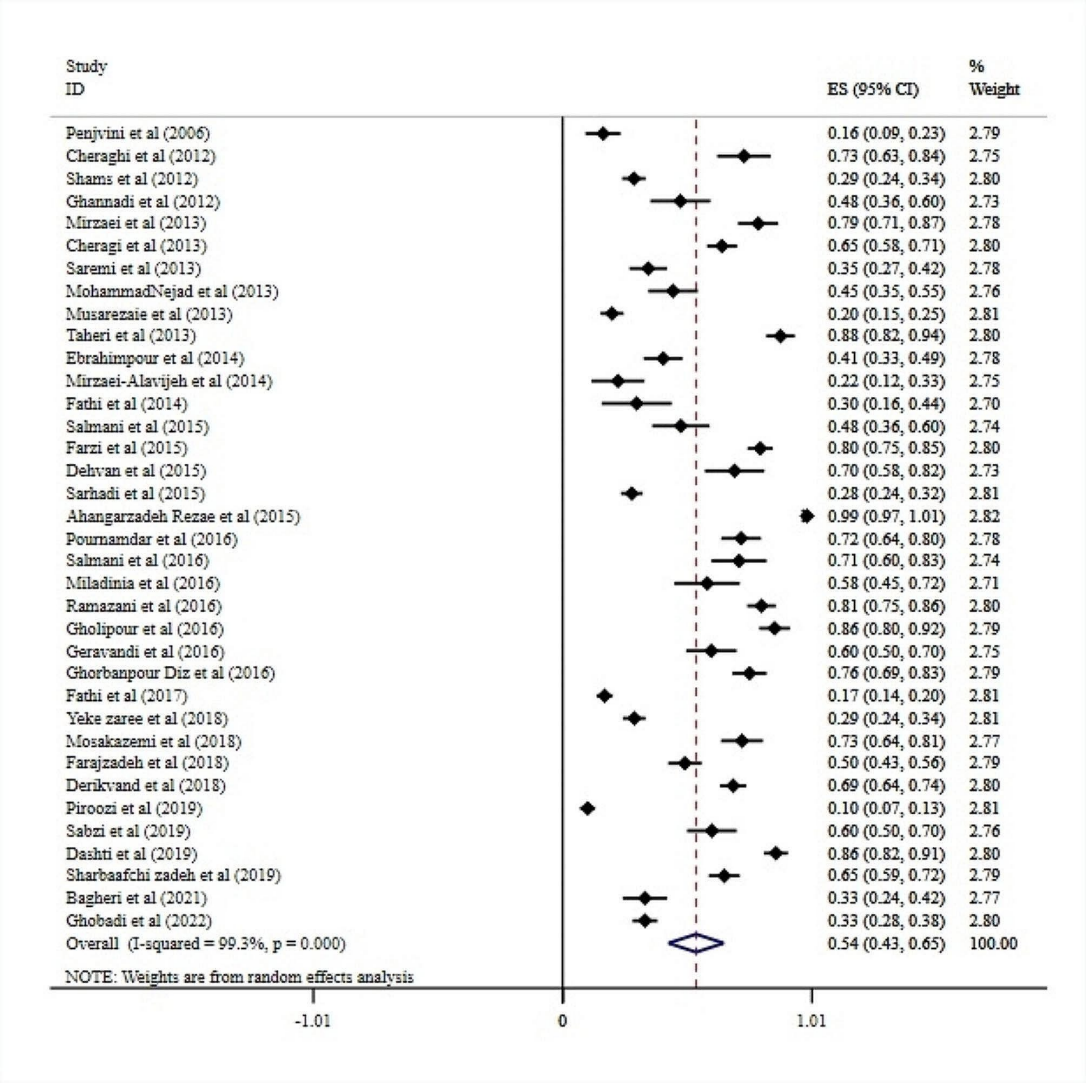
Prevalence of medication errors in nurses of Iranian hospitals (The rhombus symbol in the figure represents the overall estimate, while the small squares on each study indicate the prevalence of medication errors among nurses, and the dashed lines represent the confidence interval)

**Fig. 3:**
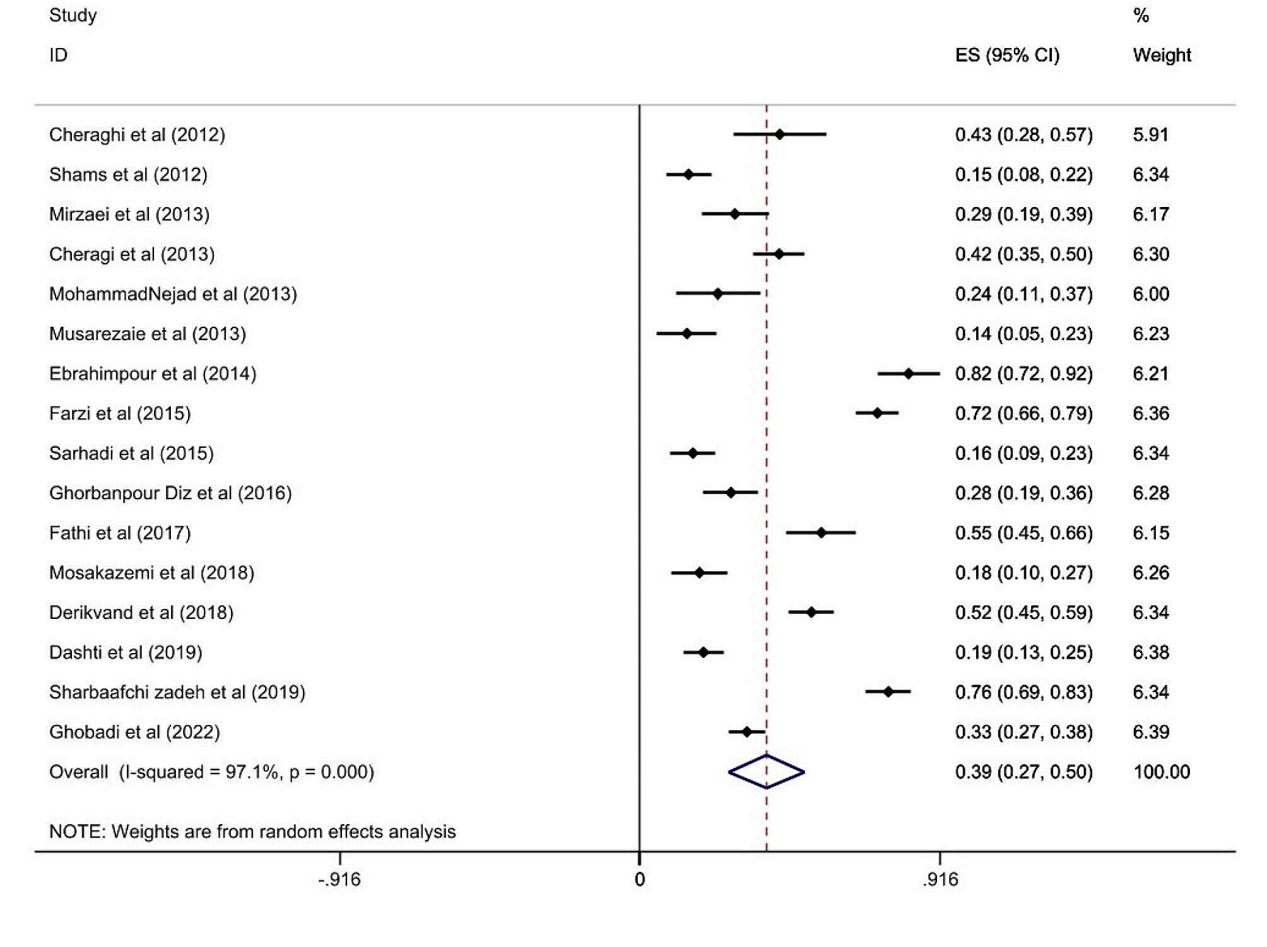
The frequency of medication error reporting among nurses (The rhombus symbol in the figure represents the overall estimate, while the small squares on each study indicate the prevalence of medication error reporting among nurses, and the dashed lines represent the confidence interval)

The prevalence of medication errors among nurses in the studies entered ranged from 10% [37]to 100% [38]. According to the results obtained from the random effects method, the prevalence of medication errors among nurses was 54% (95% CI: 43, 65; I2 = 99.3%) Fig. 2. Furthermore, the results showed that 48.5% of nurses experienced medication errors at least once, and 51.5% of them experienced two or more medication errors. According to the random effects model, unfortunately, only 39% (95% CI: 27, 50; I2 = 97.1%) of nurses report their medication errors to nursing managers Fig. 3.

In this study, the most common types of medication errors by nurses were wrong timing, accounting for 27.3% (95% CI: 19, 36; I^2^ = 95.8%), and wrong dosage, accounting for 26.4% (95% CI: 20, 33; I^2^ = 91%) Table 2. Additionally, the main causes of medication errors among nurses were workload, accounting for 43%, fatigue, accounting for 42.7%, and nursing shortage, accounting for 38.8%. The results showed that medication errors occurred more frequently during the night shift, accounting for 41%.

**Table 2:** The prevalence of Medication error according to different variables among Iranian nurses

Dependent variable		N(study)	ME	95% confidence Interval	I^2^	p-valve	Tau-squared
Type of medication error							
	wrong patient	14	17.4	12.2–22.6	87.5	0.00	0.00
	wrong medicine	15	19.7	14.8–24.6	85.2	0.00	0.008
	Wrong prescription	19	24.7	18.2–31.3	92.8	0.00	0.02
	Wrong dose	18	26.4	20.1–32.8	91.0	0.00	0.01
	wrong time	16	27.3	18.7–36	95.8	0.00	0.03
Cause of medication error							
	Fatigue	7	42.7	15.4–70.1	98.5	0.00	0.15
	Crowded workplace	7	43	18.1–67.8	98.4	0.00	0.12
	Lack of nurses	9	38.8	21.1–56.4	97	0.00	0.07
	High workload	4	16.9	5-28.9	91.3	0.00	0.01
Time of medication error							
	Morning	3	18.5	5.7–31.2	82.5	0.00	0.01
	Evening	3	25	11.6–38.3	78.6	0.00	0.01
	Night	5	41.1	33.4–48.7	54.6	0.06	0.00
Number of error reports							
	Once	6	48.5	27.5–69.6	97.1	0.00	0.06
	Twice or more	6	51.5	30-73.3	97.3	0.00	0.07
Sample size							
	< 100	13	60	44–76	98	0.0	0.08
	100–200	12	64.2	50.7–77.8	98.1	0.0	0.05
	200–300	5	53.4	33.2–73.6	98.4	0.0	0.05
	300–400	5	24.5	15.8–33.3	95.3	0.0	0.009
	> 400	1	17	13.7–20.3	-	0.0	0.000
Development of the research site							
	underprivileged	6	47.9	9.7–86.2	99.8	0.014	0.22
	Medium development	17	55	41.3–70.5	98.6	0.0	0.09
	Developed	13	56	40.6–69.8	98.6	0.0	0.07
Quality							
	Low	4	45.6	21.5–69.7	93.6	0.00	0.05
	Medium	24	62.1	51.1–73.2	98.7	0.00	0.07
	High	8	35.2	19.1–51.3	99.1	0.00	0.05
Tool							
	Researcher	20	52.6	36.2–68.9	99.4	0.00	0.13
	Other	16	56.4	41.2–71.6	99.0	0.00	0.09
Language							
	Persian	23	53.7	40.1–67.2	99.2	0.0	0.1
	English	13	55.4	38-72.7	99.1	0.0	0.1
N hospital							
	1	12	59.3	49.1–69.4		94.6	0.02
	2–5	9	56.6	39-74.2		98.2	0.07
	> 5	5	32.4	9.2–55.6		99.3	0.11

In addition to the mentioned cases, the results have shown that increasing the sample size in studies has led to a reduction in medication errors reported by nurses. Studies with a sample size below 100 participants reported 60% of medication errors, while studies with a sample size above 400 participants reported 17% of medication errors. The prevalence of medication errors decreased with an increase in the number of hospitals examined. Studies that only examined one hospital had a 59.3% prevalence of medication errors, while studies that examined more than five hospitals reported a 32.4% prevalence of medication errors. Articles that had low and moderate quality(45.6% and 62.1%) reported a significantly higher prevalence of medication errors in nurses compared to articles that had high quality(35.2%) Table 2.

The results of the meta-regression indicate a positive linear relationship between the gender ratio (female to male) in nurses and the prevalence of medication errors, as well as the year of publication Table 3. However, the relationship is inverse for the work experience and age of nurses. Fortunately, in our study, the results of sensitivity analysis showed that absence of every single study did not make a significant change in the prevalence of medication errors.

**Table 3:** Univariate meta-regression results for the prevalence of medication errors among nurses

Dependent variable	Coefficient	Standard error	p value	95% confidence interval
Mean age (years)	-0.01	0.01	0.5	-0.05 to 0.02
Mean experience (years)	-0.007	0.02	0.9	-0.05 to 0.05
Year of publish	0.004	0.01	0.7	-0.02 to 0.03
Female/men	0.01	0.01	0.07	-0.002 to 0.04

**Fig. 4:**
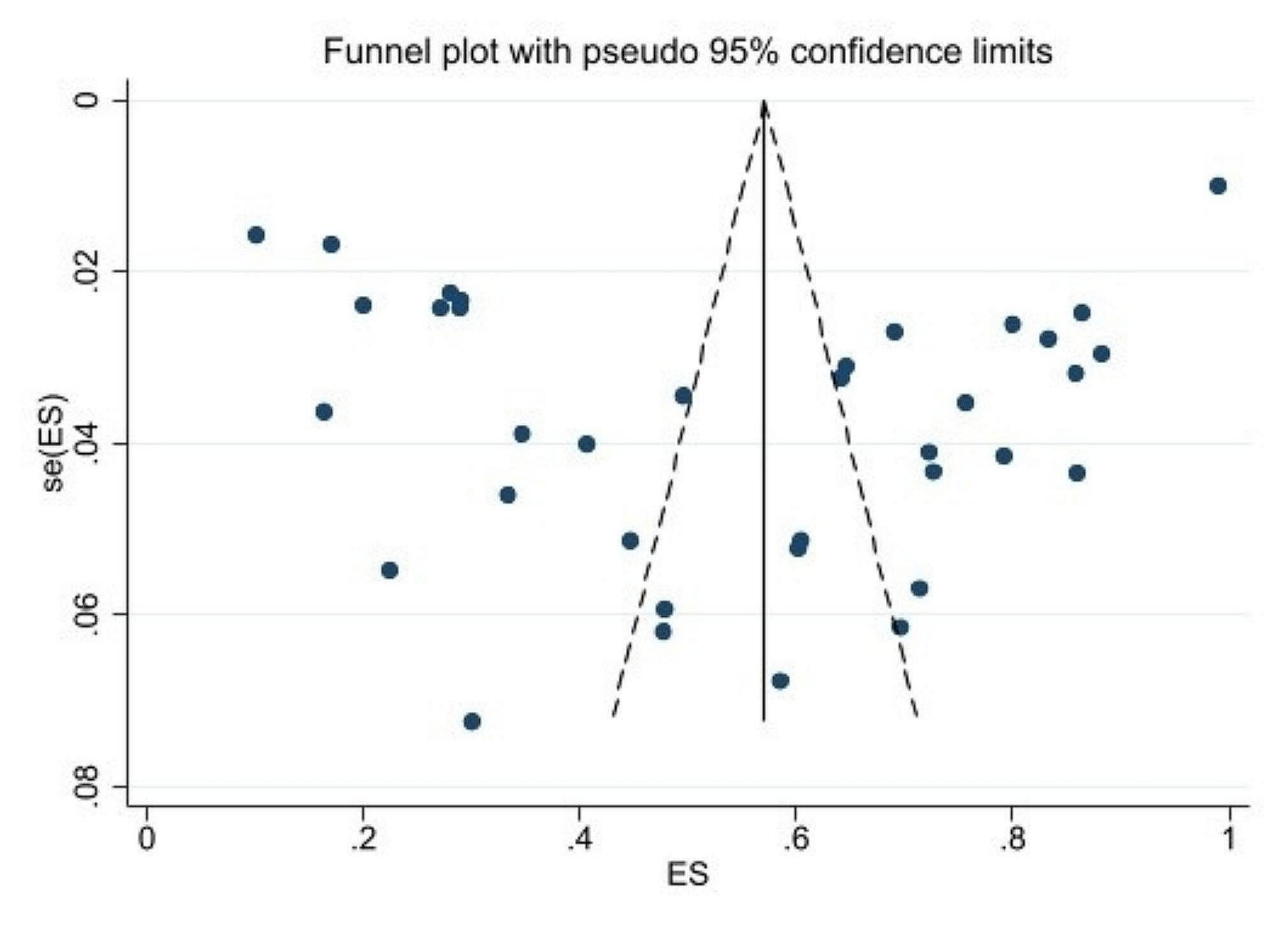
Funnel plot for assessing the risk of publication bias

To assess publication bias, funnel plots Egger’s test were employed. Each dot on the funnel plot represents a distinct study, and an uneven distribution provides evidence of publication bias [39]. The effect sizes of the studies were plotted against their standard errors, and the funnel plots were evaluated, which indicated publication bias for the prevalence of MEs as the plot appeared to be asymmetric. Although, the Begg test results (*P* = 0.733) did not indicate any signs of publication bias, the outcomes of the Egger test (*P* = 0.00) suggested the presence of such bias Fig. 4.

## Discussion

Medication errors are one of the most significant threats to patient safety in healthcare settings. This study was conducted as a systematic review and meta-analysis, and the results demonstrated that more than half of nurses commit medication errors. The study revealed that the prevalence of medication errors among Iranian nurses is 54%. This result is consistent with the results of previous studies in Iran by Sabzi (60%) [31], Farajzadeh (49.6%) [40], and Karamimatin(53%) [23]and higher than the study in Canada by Heyland (37%) [41].Possible reasons for the variations between our study and Heyland’s study may include different inclusion periods, countries, sample sizes, cultural differences, and different teachings. The occurrence of Medication errors is associated with various factors such as gender, age, work experience, hospital ward, and other related factors [42].The reason for the importance of training nurses in reducing medication errors is to enhance patient safety and improve healthcare outcomes. By providing educational programs to nurses, they can acquire the requisite knowledge and skills to proficiently manage medication errors and other medical errors, thereby improving patient safety and healthcare outcomes [43–45].

In this study, only 39% of nurses reported their medication errors, which is in line with the findings of a previous systematic review on medication errors among nurses, where only 36% of nurses reported their medication errors to nursing managers. Nurses should report errors to patients, their families, and the care team after making them, as required by professional and ethical standards. In one study [46], fear of hospital authorities was identified as a reason for not reporting nursing errors. In other study [47], nurses were stigmatized as the culprits, and in the other study [1], fear of worsening the situation was identified as a reason for not reporting nursing errors. Nursing managers can encourage nurses to report medication errors by addressing their fears and concerns and implementing management measures such as ensuring nurse anonymity. By informing other nurses, they can prevent the recurrence of errors in the future [48]. The importance of reporting medication errors for nurses should be emphasized. Reporting these errors is not only essential and necessary for enhancing patient safety in hospitals but also in all healthcare settings. Therefore, existing barriers and limitations, especially managerial barriers, should be addressed to create an environment where nurses can report errors [30].

In the field of medication errors, the most common types of errors by nurses are wrong dosage and wrong timing. In one study [32] showed that the majority of medication errors included errors in drug administration timing, drug calculation errors, dosage errors, and injection speed errors. Another study [49] showed that the most common medication errors included wrong dosage, wrong drug, and wrong timing. Due to the various dimensions and methods of investigating medication errors, the occurrence of medication errors in different studies differs from each other. Medication is the most commonly used therapeutic product in clinical settings, therefore, the frequency of its use and the necessity of its administration can increase the occurrence of errors. Furthermore, the results of this systematic review indicate that nurses attribute the highest cause of medication errors to high workload and fatigue. When nurses are confronted with multiple tasks and responsibilities, they may become overwhelmed, compromising their abilities. This can impair their ability to accurately recall medication orders and lead to errors. Nurses often work in fast-paced and time-sensitive environments. The workload pressure on nurses can result in rushing through tasks, which can contribute to errors in dosage or drug identification [50, 51].

According to the work schedule, the highest rate of medication errors occurs during the night shift. Previous studies have also confirmed these results. In fact, sleep deprivation reduces concentration and leads to an increase in errors among nurses [49, 52]. In addition, during night shifts, the occurrence of medication errors increases due to environmental factors such as reduced ambient lighting, shortage of nurses on night shifts, and fatigue and sleep deprivation [53]. Furthermore, the results demonstrated that with an increase in sample size and the number of hospitals under investigation, the occurrence of errors decreases. Increasing the sample size addresses the bias caused by a small sample size, resulting in results that are closer to reality. In other words, with an increase in sample size, individuals from different age groups, genders, and occupational groups are present [54].

The results of the regression analysis in this study demonstrated that increasing age and work experience are associated with a decrease in medication errors. Younger nurses with less work experience are more prone to medication errors due to lower practical skills, limited experience in high-pressure environments, high job stress in the early years of their careers, and higher workload compared to more experienced nurses in Iran [4, 6]. Older nurses, on the other hand, possess higher levels of skill and extensive experience, often assuming managerial roles and having less direct contact with patients and medical devices, which reduces the occurrence of errors in their practice [55]. Another part of the regression analysis results indicates that women report a higher proportion of medication errors compared to men, which is consistent with the findings [23].

The results of the funnel plot showed asymmetry in the published results, which was confirmed by the Egger test. It should also be noted that the present study only examined observational studies and relied on self-reporting, which may differ from actual results. This review study has some limitations that need to be considered for publication in scientific journals. Our study only examined Iranian nurses and did not include other nurses from different countries or other healthcare workers, which makes it impossible to generalize the results. Furthermore, our study does not have the ability to identify the causes of medication errors, the outcomes of medication errors, and events following medication errors.

## Implications for research and practice

This study demonstrates that the prevalence of medication errors in Iran is significantly high, underscoring the importance of identifying predictive factors for medication errors [56]. Therefore, further research is needed to uncover the underlying causes of medication errors among Iranian nurses, particularly in relation to workload, fatigue, and nurse shortages. Identifying the root causes of these errors can aid in the development of targeted interventions to prevent them. Additionally, future studies should focus on interventions aimed at reducing medication errors among nurses, such as implementing medication safety protocols, providing adequate training and support, and promoting a culture of error reporting without fear of consequences [57]. To achieve this, healthcare systems in Iran must prioritize creating a safe and supportive work environment for nurses with sufficient staffing levels and resources to prevent medication errors [58].

## Conclusions

In this systematic review, we have found that more than half of Iranian nurses suffer from medication errors, and most nurses who experience medication errors do not report their errors to nursing managers. Hospital administrators should provide a calm and stress-free environment to reduce medication errors and alleviate the busyness of hospital wards and nurses’ workload. Additionally, hospital administrators should support nurses who report their errors and implement appropriate measures for key factors contributing to errors, such as night shifts.

### Electronic supplementary material

Below is the link to the electronic supplementary material.

Supplementary Material 1

Supplementary Material 2

## Data Availability

The datasets used and/or analyzed during the current study are available from the corresponding author upon reasonable request.
