# Supplementary material for: Prevalence of medication errors and its related factors in Iranian nurses: an updated systematic review and meta-analysis
Source: BMC Nurs. 2024 Mar 14;23:175. doi: 10.1186/s12912-024-01836-w (PMC10938711; doi:10.1186/s12912-024-01836-w)
Supplement: Supplementary file 1 — Supplementary Material 1 [file 12912_2024_1836_MOESM1_ESM.docx]

Prevalence of medication errors and its related factors in Iranian nurses: an updated systematic review and meta-analysis

*Hadis Fathizadeh^1^, Samaneh Mosavi^2^ ,Zahra Gharibi^3^,Hamidreza Rezaeipour^2^ ,**Abdol rahim biojmajd^4^*

*1-* *Department of laboratory sciences, Sirjan school of medical sciences, Sirjan, Iran.*

*2-* *Student Research Committee, Sirjan School of Medical Sciences, Sirjan, Iran.*

*3-* *Infectious and Tropical Diseases Research Center, Hormozgan Health Institute, Hormozgan University of Medical Sciences, Bandar Abbas, Iran.*

*4- Department of Nursing, Sirjan School of Medical Sciences, Sirjan, Iran.*

| Quality | Q8 | Q7 | Q6 | Q5 | Q4 | Q3 | Q2 | Q1 | First author |
| --- | --- | --- | --- | --- | --- | --- | --- | --- | --- |
| Medium | Y | U | Y | L | L | Y | U | Y | Salmani et al |
| Medium | Y | Y | Y | L | L | Y | Y | Y | Cheraghi et al |
| Medium | Y | U | Y | L | U | Y | U | Y | Mirzaei et al |
| Medium | U | Y | Y | L | Y | Y | U | Y | Pournamdar et al |
| High | Y | Y | Y | Y | Y | U | Y | Y | Fathi et al |
| Low | L | L | U | Y | Y | L | L | L | Salmani et al |
| Medium | L | Y | U | L | L | Y | Y | Y | Cheragi et al |
| High | L | L | Y | L | U | Y | Y | Y | Piroozi et al |
| Low | U | U | L | U | U | L | L | L | Miladinia et al |
| High | Y | Y | Y | Y | Y | Y | Y | Y | Yeke zaree et al |
| Medium | Y | Y | Y | L | U | U | Y | Y | Saremi et al |
| Medium | Y | Y | Y | L | U | Y | U | Y | Mohammad-Nejad et al |
| High | Y | Y | Y | Y | U | Y | Y | Y | Shams et al |
| High | Y | Y | Y | U | Y | Y | Y | Y | Musarezaie et al |
| Medium | U | Y | Y | L | L | Y | U | Y | Ebrahimpour et al |
| Medium | U | Y | Y | L | U | Y | Y | Y | Taheri et al |
| Medium | Y | Y | Y | U | U | Y | U | Y | Ramazani et al |
| Medium | Y | Y | Y | U | L | L | U | Y | Gholipour et al |
| High | Y | U | Y | Y | Y | Y | Y | Y | Farzi et al |
| Medium | Y | U | Y | L | L | Y | Y | Y | Mosakazemi et al |
| Medium | Y | Y | Y | L | L | Y | U | Y | Farajzadeh et al |
| Medium | U | Y | Y | U | U | L | Y | Y | Geravandi et al |
| Medium | Y | Y | Y | L | U | U | U | Y | Sabzi et al |
| Low | Y | Y | L | U | U | L | L | L | Mirzaei-Alavijeh et al |
| Medium | Y | Y | Y | L | L | Y | Y | Y | Bagheri et al |
| Medium | Y | Y | U | U | L | Y | L | Y | Dehvan et al |
| Medium | Y | Y | Y | U | U | Y | L | L | Dashti et al |
| Medium | Y | Y | U | L | U | Y | Y | Y | Sharbaafchi zadeh et al |
| High | U | Y | Y | Y | Y | Y | Y | Y | Sarhadi et al |
| Medium | Y | Y | Y | U | L | Y | Y | Y | Ahangarzadeh Rezae et al |
| Medium | Y | Y | L | Y | L | Y | L | Y | Ghorbanpour Diz et al |
| High | Y | Y | U | Y | Y | Y | Y | Y | Derikvand et al |
| Low | L | L | U | Y | U | L | L | Y | Fathi et al |
| Medium | L | Y | Y | L | L | Y | Y | Y | Penjvini et al |
| Medium | Y | L | Y | Y | L | Y | Y | Y | Ghannadi et al |
| Medium | L | Y | Y | L | L | Y | L | Y | Ghobadi et al |

Table S1: Quality assignments based on the JBI(Joanna Briggs Institute)

| Not  Applicable | No | Yes | Criteria | no |
| --- | --- | --- | --- | --- |
|  |  |  | Were the criteria for inclusion in the sample clearly defined? | 1 |
|  |  |  | Were the study subjects and the setting described in detail? | 2 |
|  |  |  | Was the exposure measured in a valid and reliable way? | 3 |
|  |  |  | Were objective, standard criteria used for measurement of the condition? | 4 |
|  |  |  | Were confounding factors identified? | 5 |
|  |  |  | Were strategies to deal with confounding factors stated? | 6 |
|  |  |  | Were the outcomes measured in a valid and reliable way? | 7 |
|  |  |  | Was appropriate statistical analysis used | 8 |

Table S2: JBI quality measurement tools
